# Mucosal adherent bacterial dysbiosis in patients with colorectal adenomas

**DOI:** 10.1038/srep26337

**Published:** 2016-05-19

**Authors:** Yingying Lu, Jing Chen, Junyuan Zheng, Guoyong Hu, Jingjing Wang, Chunlan Huang, Lihong Lou, Xingpeng Wang, Yue Zeng

**Affiliations:** 1 Department of Gastroenterology, Shanghai general Hospital, Shanghai JiaoTong University School of Medicine, Shanghai, 200080, China; 2 International Medical Care Center, Shanghai general Hospital, Shanghai JiaoTong University School of Medicine, Shanghai, 200080, China

## Abstract

Recent reports have suggested that the gut microbiota is involved in the progression of colorectal cancer (CRC). The composition of gut microbiota in CRC precursors has not been adequately described. To characterize the structure of adherent microbiota in this disease, we conducted pyrosequencing-based analysis of 16S rRNA genes to determine the bacterial profile of normal colons (healthy controls) and colorectal adenomas (CRC precursors). Adenoma mucosal biopsy samples and adjacent normal colonic mucosa from 31 patients with adenomas and 20 healthy volunteers were profiled using the Illumina MiSeq platform. Principal coordinate analysis (PCoA) showed structural segregation between colorectal adenomatous tissue and control tissue. Alpha diversity estimations revealed higher microbiota diversity in samples from patients with adenomas. Taxonomic analysis illustrated that abundance of eight phyla (Firmicutes, Proteobacteria, Bacteroidetes, Actinobacteria, Chloroflexi, Cyanobacteria, Candidate-division TM7, and Tenericutes) was significantly different. In addition, *Lactococcus* and *Pseudomonas* were enriched in preneoplastic tissue, whereas *Enterococcus*, *Bacillus*, and *Solibacillus* were reduced. However, both PCoA and cluster tree analyses showed similar microbiota structure between adenomatous and adjacent non-adenoma tissues. These present findings provide preliminary experimental evidence supporting that colorectal preneoplastic lesion may be the most important factor leading to alterations in bacterial community composition.

Colorectal cancer (CRC) is one of the leading contributors to cancer-related deaths. It is the fourth most commonly diagnosed cancer worldwide, with more than 1 million new cases diagnosed annually, and its incidence has increased rapidly in recent years in China^1^. Most sporadic CRC begins with the formation of polyps and is preceded by dysplastic adenomas, which can progress into malignant forms. This process is referred to as the adenoma-carcinoma sequence that occurs through a multistep mechanism associated with mutations in the adenomatous polyposis coli (APC) gene and in components of the mitogen-activated protein kinase (MAPK) signaling pathway, such as KRAS^2^.

The human gastrointestinal tract is colonized by complex and diverse communities of commensal microorganisms^3^. The total number of bacterial species in the gastrointestinal tract is about 10-fold greater than that of human cells in the body^4^. These commensal bacteria contribute to regulation of cell proliferation, differentiation, and gene expression in host epithelial cells through downstream signaling pathways^5^. The microbiota impacts numerous physiological functions related to cancer risk, and a substantial amount of research has confirmed that the gut microbiota is a primary driver of inflammation in the colon and is linked to CRC development. Some studies have shown evidence of microbial dysbiosis between patients at various stages of CRC and healthy controls. Sobhani *et al.* reported that the genus group *Bacteroides*-*Prevotella* is over-represented in CRC patients compared with normal subjects^6^. Wang *et al.* demonstrated a decreased abundance of butyrate-producing bacteria in CRC patients^7^. Thus far, many studies strongly suggest that *Fusobacterium* may be associated with CRC^8^,^9^, although more functional analysis may be necessary to gain a better understanding of the role of microorganisms in CRC. As for colorectal adenoma, the precursor to CRC, no relatively specific bacterial species has been identified as a risk factor. In an American patient cohort, an increased abundance of *Firmicutes*, *Bacteroidetes*, and *Proteobacteria* was observed, compared with non-adenoma subjects, in adenoma biopsies^10^. However, it is still unknown if the gut microbiota plays a role in the early stages of colorectal carcinogenesis.

In this study, we performed a microbiome analysis of colorectal mucosal biopsies from healthy volunteers and polyp patients (adenomatous polyp tissue and matched normal adjacent tissue) to elucidate bacterial changes that might induce or accompany early oncogenic transformation of CRC using a deep sequencing platform.

## Results

### Characteristics of pyrosequencing results

In total, 1180268 usable sequences were obtained from 82 samples using pyrosequencing. From these, 1138898 high-quality sequences were selected, with an average of 13889 sequences per sample. In total, 2083 OTUs were delineated at a 97% similarity level. The values of Good’s coverage of all libraries were above 99%. The rarefaction curves did not approach a plateau with the current sequencing, but the Shannon diversity estimates of all the samples were stable, suggesting that although new phylotypes would be expected with additional sequencing, most diversity had already been captured (Figs S1 and S2). Other diversity estimators of community are shown in Table 1. There were statistically significant differences in Shannon indices, Simpson indices, Chao I, ACE indices, and OTU numbers between volunteers and patients (Student’s *t*-test; ^**^P < 0.05; ***P < 0.01), demonstrating a significantly higher diversity found in patients with colorectal adenomas compared with healthy control tissue. The characteristics of each sample are shown in Table S1.

### Structural comparisons of tissue microbiota between healthy volunteers and patients with colorectal adenoma

**Figure 1:**
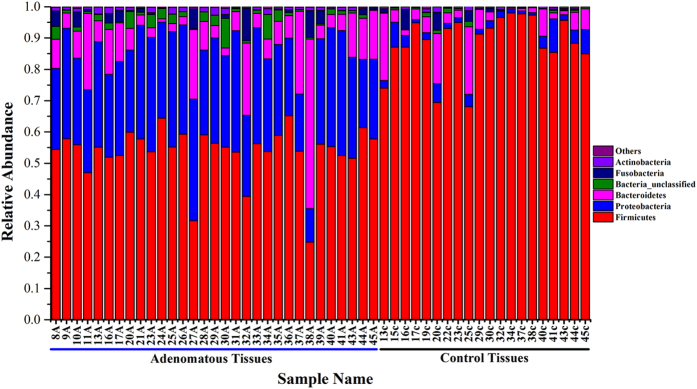
Relative abundance of bacterial phyla in microbiota of two groups of samples. A capital ‘A’ indicates adenomatous tissue; a lowercase ‘c’ indicates control tissue from healthy volunteers.

**Figure 2:**
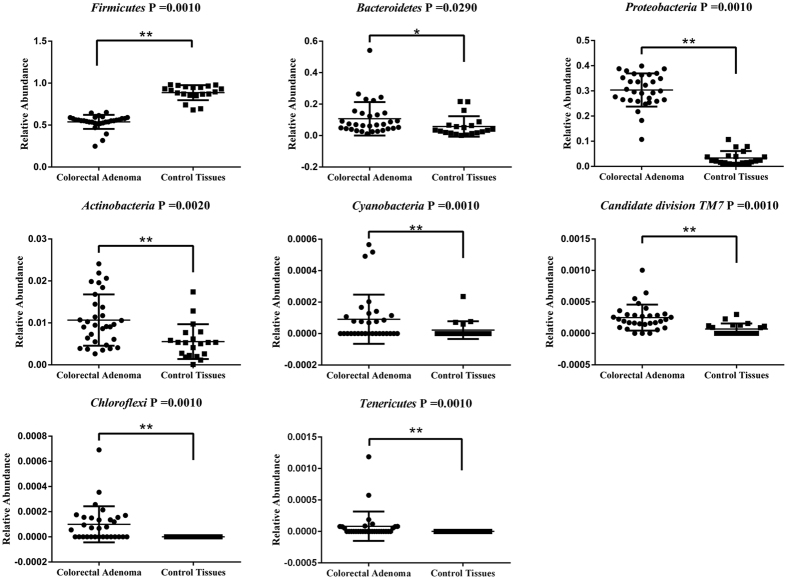
Relative abundance of significantly different phyla between adenomatous and control tissues.

Nineteen phyla were revealed from 31 colorectal adenomatous tissues and 20 healthy tissues. The bacteria flora analysis showed that Firmicutes was the most predominant phylum, contributing 88.6% and 53.7% of the tissue microbiota in healthy volunteers and colorectal adenoma patients, respectively. The second and third most dominant phyla in colorectal adenoma patients were Proteobacteria (30.1%) and Bacteroidetes (10.8%). However, there was a clear decrease in both of these phyla (Proteobacteria 3.3%, Bacteroidetes 5.8%) in healthy volunteers compared with patients with colorectal adenoma patients (Fig. 1). Metastat analysis indicated that eight phyla, Firmicutes, Proteobacteria, Bacteroidetes, Actinobacteria, Chloroflexi, Cyanobacteria, Candidate-division TM7, and Tenericutes, were significantly different between colorectal adenomatous tissue and healthy tissue (Fig. 2).

**Figure 3:**
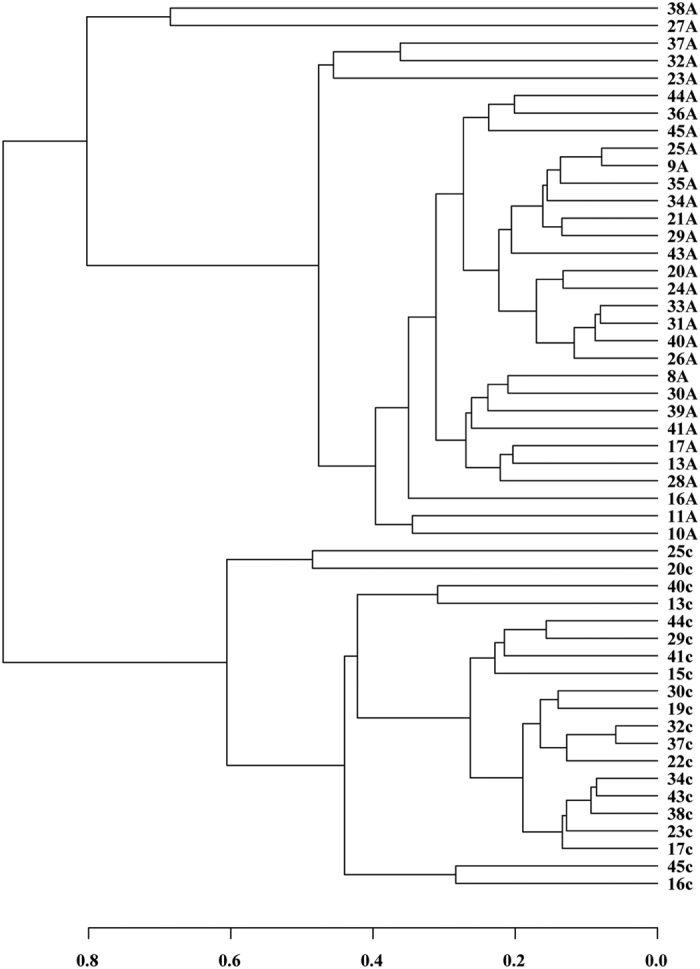
Cluster tree based on Bray-Curtis distances among three groups.

**Figure 4:**
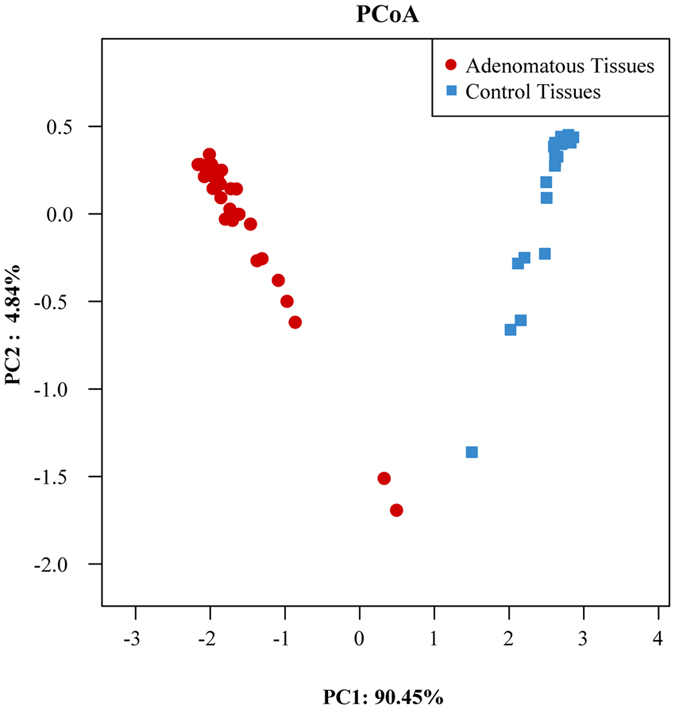
PCoA plots based on Bray-Curtis metrics between adenomatous and control tissues. Each symbol represents one sample.

Beta diversity of each group was calculated through cluster tree analysis and PCoA and Non-metric multidimensional analysis(NMDS). Cluster tree analysis indicated that the bacterial structure was obviously separated between colorectal adenomatous and healthy tissues, and the bacterial composition of the same group was more similar (Fig. 3). PCoA based on the relative abundance of OTUs revealed a separation of the healthy and colorectal adenomatous tissues based on the first two principal component scores, which accounted for 90.45% and 4.84% of the total variations, suggesting that disease may be one of the important factors accounting for the change in structure (Fig. 4). NMDS of bacterial community also showed clear separation (Fig. S4).

**Figure 5:**
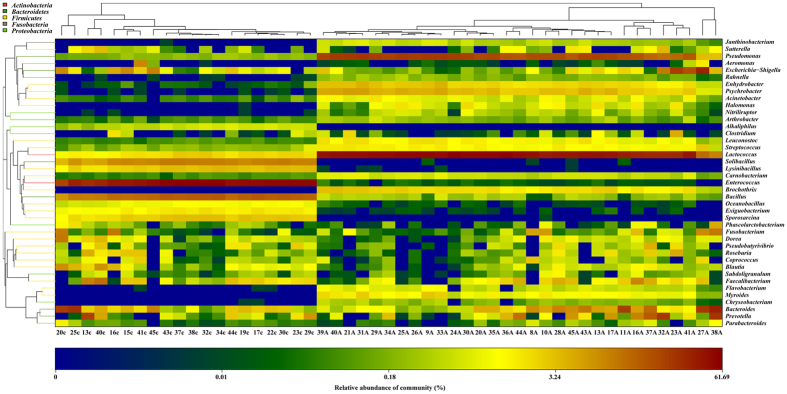
Relative abundance of the genera in the differentiation of microbiota of healthy volunteers and patients with adenoma.

**Figure 6:**
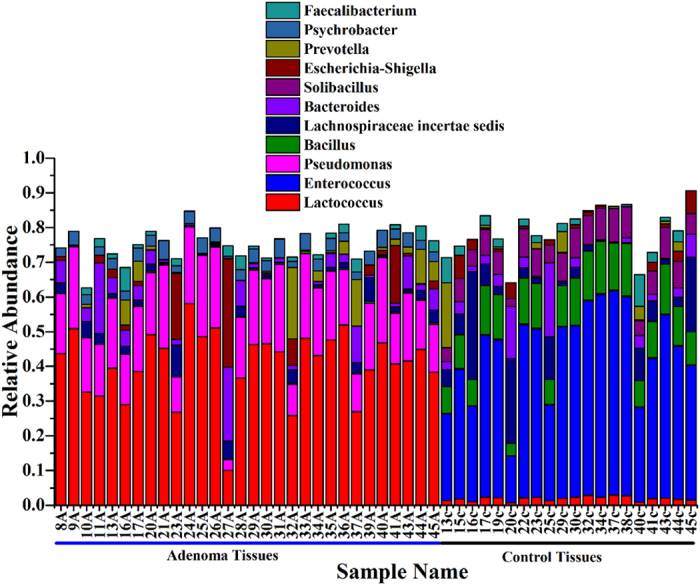
Relative abundance of bacterial genera in microbiota of healthy volunteers and patients with adenoma.

**Figure 7:**
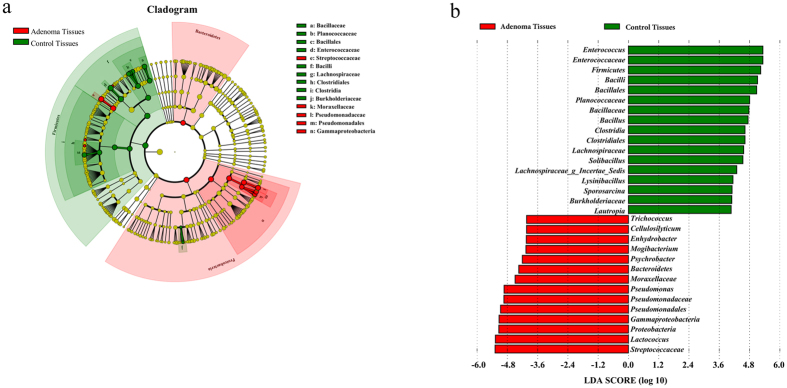
Different structures of adenomatous and control tissue microbiota. (**a**) Taxonomic representation of statistically and biologically consistent differences between adenomatous and control tissues. Differences are represented by the color of the most abundant class (red indicates adenomatous tissue; yellow, non-significant; and green, control). The diameter of each circle is proportional to taxon abundance. (**b**) Histogram of the linear discriminant analysis (LDA) scores for differentially abundant genera.

More than 200 genera were divided through the SILVA database. The top 42 dominant genera in all samples were selected to construct a heat map, which reflected that all 42 genera belong to five main phyla: Proteobacteria, Bacteroidetes, Firmicutes, Actinobacteria, and Fusobacteria. The dominant genera between colorectal adenomatous and healthy tissues were different. Most of the dominant genera in healthy tissue belonged to Firmicutes and most of the dominant genera in colorectal adenomatous tissue belonged to Proteobacteria (Fig. 5). The sequences that were classified into the 11 dominant genera accounted for more than 60% of the total sequences. *Lactococcus* was the predominant genus in colorectal adenomatous tissue, and the content was much higher than in healthy tissue. The second dominant genus in colorectal adenomatous tissue was *Pseudomonas*, whose content in healthy tissue was less than 0.1%. Conversely, the dominant genera in healthy tissue were *Enterococcus* and *Bacillus*, the contents of which were much higher than those in colorectal adenomatous tissue. In addition, another dominant genus, *Solibacillus*, in healthy tissue was virtually absent in colorectal adenomatous tissue (Fig. 6). The greatest differences in taxa between the two communities were displayed using LEfSe (Fig. 7).

### Comparison of bacterial communities of colorectal adenomatous and adjacent tissues

**Figure 8:**
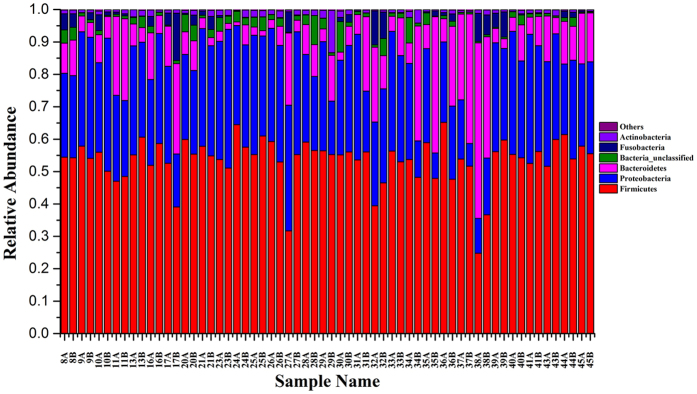
Relative abundance of bacterial phyla in the microbiota of colorectal adenomatous and adjacent tissues.

**Figure 9:**
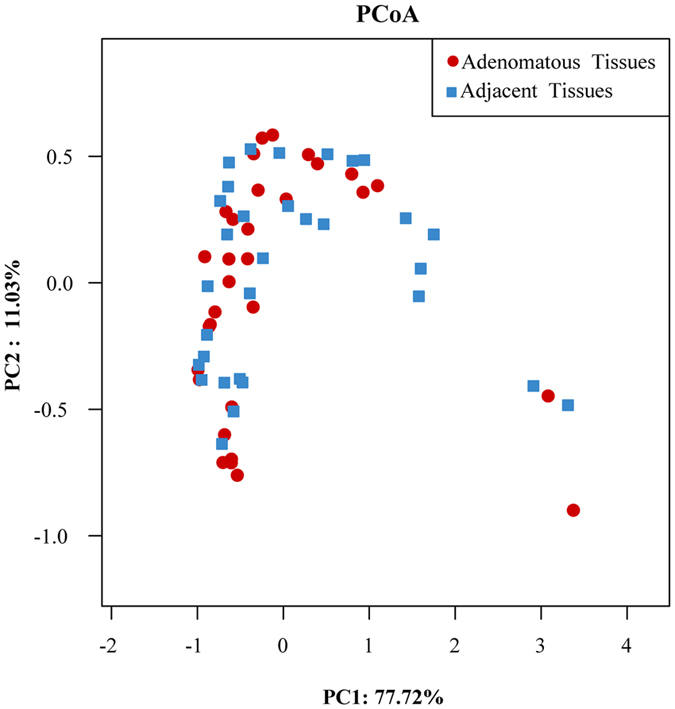
PCoA plots based on Bray-Curtis metrics between colorectal adenomatous and adjacent tissues.

The bacterial communities were compared between colorectal adenomatous and adjacent tissues in the same patient. Firmicutes was the most predominant phylum contributing about 50% in two tissues. The following dominate phylum was Proteobateria and Bacterodetes with the proportion of about 27.32% and 13.69% in two group tissues, respectively. The bacterial communities of the two tissues from one patient were not remarkably different (Fig. 8). The difference between adenomatous and adjacent tissues is shown by a Venn diagram (Fig. S3). Seven hundred and sixty-six OTUs were shared between the two tissues, indicating that colorectal adenomatous and adjacent tissues were more similar in terms of OTU level. PCoA analysis based on the OTU information of each sample indicated that there were no significant differences in the bacterial composition between these two tissues from the same patient (Fig. 9).

## Discussion

The human intestinal tract is inhabited by a complex community of microorganisms. Increasing evidence indicates that the intestinal microbial community plays an important role in the pathogenesis of the progression from normal to advanced adenoma and subsequently to CRC. Many studies have consistently demonstrated significant differences in the microbial community structure of patients with CRC^11^, and one study showing the emergence of CRC-associated microorganisms was mainly focused on *Fusobacterium nucleatum*^12^. However, studies on the specific gut microbiome composition and profile associated with CRC or advanced adenoma have shown inconsistent results. As we know, adherent bacteria might be more prone to affect gene expression in colon mucosal cells than transient bacteria that are expelled in the feces. In this study, we explored the profile of the intestinal mucosal-associated microbiota in 31 subjects with adenomas (adenoma biopsy samples and adjacent normal tissue samples) and 20 healthy controls by using the Illumina MiSeq platform. The bacterial diversity and richness indices in patients with advanced adenoma were significantly higher than those in the healthy control subjects. This finding was not unexpected because Qiang *et al.* reported increased gut microbiome richness in CRC by a metagenome-wide association study on stools from advanced adenoma or carcinoma patients and from healthy subjects^13^.

Numerous laboratories have compared the microbiomes of patients at various stages of CRC with those of healthy controls and found evidence of microbial dysbiosis. As a CRC precursor, colorectal adenomas have become increasingly important in the study of colorectal carcinogenesis. As we know, the Firmicutes/Bacteroidetes ratio is considered representative of health status, and may reflect the eubiosis of the gastrointestinal tract. In the present study, our observation that Firmicutes, Proteobacteria, and Bacteroidetes were the dominant phyla in healthy volunteers are also concordant with previous studies of the gut microflora^14^. However, a large decrease in Firmicutes with concomitant relative expansion of Proteobacteria was observed in patients with adenomas. Proteobacteria, which was more abundant in patients with adenomas, are generally regarded as gut commensals with potential pathogenic features^15^. In this study, we found that Chloroflexi and Tenericutes were significantly enriched in adenoma biopsy samples compared with control group samples, in which these two phyla were barely detected. Interestingly, the major differences between the two groups including Chloroflexi, Tenericutes, and Candidate-division TM7 were the common resident oral microbiota^16^. In contrast, patients had a corresponding decrease in the Firmicutes/Bacteroidetes ratio, which could be considered an important marker for intestinal dysbiosis of colorectal precancerous lesions.

In our study, at the genus level, we observed statistically significant differences in bacterial abundance between patients and healthy volunteers. Our PCoA was based on the first two principal component scores, one of which accounted for 90.45%, which indicates that disease (colorectal adenoma) is the most important factor contributing to changes in structure, considering that controls were matched for age and gender, had similar diets, and received no recent medication. In other words, the development of adenomas may contribute to microbiota imbalances. Mutually, local microbiota disturbances may further promote precancerous lesions. Disturbance in the balance between adenomas and the microbiota, whether causal or consequential, may still be a problem and requires further study to elucidate their relationship. In particular, we found that increased relative abundance of potential opportunistic pathogens such as *Pseudomonas* and *Streptococcus*, which contribute to changes in intestinal homeostasis, might display robust inflammatory infiltration and directly or indirectly increase the risk of adenoma development. Vázquez-Rivera *et al.* showed that toxins secreted by *Pseudomonas aeruginosa* can promote cell death in cultures of Caco-2 colorectal adenocarcinoma cell lines *in vitro*^17^. *Streptococcus bovis* promotes the progression of pre-neoplastic lesions by increasing cell proliferation and interleukin-8 production in a rat model^18^. *Lactococcus*, which is generally regarded as a gut commensal with probiotic features, was found to be the predominant genus in adenomatous tissue, suggesting that the microbial shifts are caused by quite dramatic physiological alterations that result from colon carcinogenesis itself^19^. We propose that local changed gut environment may favor increased abundance of specific taxa. *Lactococcus* was identified as a potential passenger bacterium that is capable of remodeling the mucosal gut environment according to driver-passenger model^20^. Interestingly, *Solibacillus*, a new member of the genus *Bacillus* and about which little is known regarding its clinical epidemiology and pathogenicity in CRC development, was found to be significantly over-represented in our patients. Future studies involving gnotobiotic animal models should be utilized to elucidate the mechanisms by which *Solibacillus*-altered colonic bacterial profiles might be associated with adenoma progression.

In our study, we found *blautia* was reduced in patients with adenomas which was consistent with the previous report in CRC patients^21^. In contrast, we observed no differences in the abundance of *Akkermansia*, or *Fusobacterium* which have been reported to correlate with CRC between healthy controls and advanced adenoma samples^9^,^21^,^22^. As we know, *Akkermansia* is known to break down mucin, while mucin degradation has been linked to intestinal inflammation and can facilitate colonization of intestinal pathogens^23^. We also found no differences in butyrate-producing bacteria (*Clostridium*, *Roseburia*), or *Faecalibacterium* or *Bifidobacterium* whereas a previous study showed depletion of butyrate-producing bacteria, *Faecalibacterium* or *Bifidobacterium* in CRC patients^7^,^21^,^24^,^25^,^26^. Future clinical analyses taking into account factors such as obesity, alcohol, race, and meal time would help resolve the possible roles of these conflicting results.

Previous studies have indicated that there are obvious differences in community composition between cancerous tissues and surrounding areas among late-stage CRC patients. These findings led to a bacterial driver-passenger model for colorectal cancer^20^. Shen *et al.* observed changes in rectal mucosal bacterial communities of adenoma patients as well as in healthy controls, and hypothesized that rectal mucosal bacterial composition may reflect the presence of adenoma bacterial communities^27^. However, few studies have directly investigated the bacterial composition of adenomatous tissues. Specifically, in our study, we compared adenoma biopsy samples with their adjacent non-adenoma samples from the same patient. However, there were no significant abundance differences across any of the sampled tissues, indicating that the driver-passenger hypothesis may not be fully relevant to the precancerous colon lesion. The overall microbial structures of precancerous and adjacent tissues were similar, unlike one other CRC-related study^28^. Microbiome structural dysbiosis, which likely leads to a changing pro-oncogenic microenvironment, indicates that different disease states have a specific microbial signature^29^. Thus, future studies may help identify the link between bacterial composition shifts with different stages of colorectal cancer development and resolve this paradox. More research is required to reveal the correlation between changes in the intestinal microenvironment and microbiota homeostasis during a process from normal tissue to hyperplasia, adenoma and eventually carcinoma.

In conclusion, by comparing the intestinal microbiota composition between adenoma patients and healthy individuals, we have defined a structural imbalance in the gut microbiota, represented by an increased incidence of opportunistic pathogens in patients and reduction of *Solibacillus*, a new member of the genus *Bacillus*, with little information available concerning the role of probiotics on colorectal carcinogenesis. PCoA analysis suggested that colorectal pre-neoplastic lesion is the primary factor leading to changes in the adherent bacterial structure. However, we also found no differences between adenomatous and adjacent mucosal microbiota composition, which indicates that local mucosal epithelium transits to a pro-inflammatory microenvironment that is conducive to colorectal tumorigenesis. Identifying the composition of adherent bacterial communities in the precancerous colon is an important step in our development of effective diagnostic, preventive, or therapeutic strategies.

## Methods

### Sampling

Thirty-one patients with colon adenomas, aged 27–67 years, were selected from the Shanghai General Hospital from Jan 1 to Jun 30, 2014. All patients were of independent genetic background. Exclusion criteria included pregnancy, colitis (either ulcerative, Crohn’s, radiation-induced, or infectious colitis or other chronic inflammatory illnesses), polyposis (>100 polyps), previous colon resection, or colorectal cancer. Twenty healthy volunteers were selected in the Shanghai General Hospital as controls after a routine physical examination. No subjects were taking medications at the time of sample collection or had used antibiotics in the past 3 months before sample collection (Table 2). All samples were collected in accordance with the relevant guidelines and regulations, and the research was approved by the Research Ethics Boards of Shanghai General Hospital. Informed consent was provided by all individuals. All patients received standard instructions to prepare for colonoscopy. Samples from one adenomatous tissue and its adjacent non-adenomatous tissue (5 cm regions from the location of the adenomas) were collected from each patient by biopsy forceps during colonoscopy, and one tissue sample from the same position was collected from healthy volunteers as control. All samples were frozen immediately after sampling and stored at −80 °C.

### DNA extraction and PCR amplification

Microbial genomic DNA was extracted from each samples using the E. Z. N. A.^®^ Soil DNA Kit (Omega Bio-tek, Norcross, GA, U.S.) according to manufacturer’s protocols. Samples were placed into bead tubes with 500 mg of glass beads. Add 1 ml Buffer of SLX Mlus to the samples. Incubate at 70 °C for 10 min. Bead beating at 6.5 m/s for 5 min using a FastPrep-24 bead beater (MP Biomedicals, Santa Ana, USA) . Total DNA was eluted in 60 μL of Elution buffer. The amount of DNA was determined by NanoDrop2000 (Thermo Scientific). Integrity and size of DNA were checked by 1% (wt/vol) agarose gel electrophoresis in 0.5 mg/ml ethidium bromide. All DNA samples were stored at −20 °C until further processing. The V3-V4 region of the bacteria 16S ribosomal RNA gene from each sample were amplified using the bacterial universal primer 338F 5′-barcode-ACTCCTACGGGAGGCAGCA-3′ and 806R 5′-barcode-GGACTACHVGGGTWTCTAAT-3′^30^, where barcode is an six to eight-base sequence unique to each sample. PCR reactions were performed in triplicate 20 μL mixture containing 4 μL of 5× FastPfu Buffer, 2 μL of 2.5 mMdNTPs, 0.8 μL of each primer (5 μM) (Sangon Biotech, Shanghai), 0.4 μL of FastPfu Polymerase (TransStart^®^ FastPfu DNA Polymerase, TransGen BioTech, Beijing, AP221-01), and 10 ng of template DNA. PCR was performed on Thermal Cycler (Bio-Rad, USA) with following procedure, 95 °C for 3 min, followed by 25 cycles at 95 °C for 30 s, 55 °C for 30 s, and 72 °C for 30 s and a final extension at 72 °C for 5 min. The PCR products were evaluated using 2% (wt/vol) agarose gel electrophoresis.

### Pyrosequencing

Amplicons were extracted from 2% agarose gels and purified using the AxyPrep DNA Gel Extraction Kit (Axygen Biosciences, Union City, CA, U.S.) according to the manufacturer’s instructions and quantified using QuantiFluor™ -ST (Promega, U.S.). Purified amplicons were pooled in equimolar. Index PCR and sequencing according to the Illumina MiSeq 16S Metagenomic Sequencing Library Preparation protocol (http://support.illumina.com/downloads/16s_metagenomic_sequencing_library preparation.html). Briefly, Samples were multiplexed using a dual-index approach with the Nextera XT Index kit (Illumina Inc., San Diego, CA, USA) according to the manufacturer’s instructions. The final library was paired-end sequenced at 2 × 250 bp using a MiSeq Reagent Kit v2 on the Illumina MiSeq platform.

Raw fastq files were demultiplexed and quality-filtered using Trimmomatic^31^ and FLASH^32^ software with the following criteria: (I) the 300-bp reads were truncated at any site receiving an average quality score of <20 over a 50-bp sliding window, discarding the truncated reads that were shorter than 50 bp; (II) exact barcode matching was required, 2-nucleotide mismatch in primer matching and reads containing ambiguous characters were removed; and (III) only sequences that overlapped by more than 10 bp were assembled according to their overlap sequence. Reads that could not be assembled were discarded.

### Data Availability

The 16S sequence data generated in this study were submitted to the GenBank Sequence Read Archive accession number SRP064975.

### Bioinformatics Analysis

The high-quality sequences were assigned to samples according to barcodes. Operational taxonomic units (OTUs) were clustered with 97% similarity cutoff using UPARSE (version 7.1 http://drive5.com/uparse/) and chimeric sequences were identified and removed using UCHIME. The taxonomy of each 16S rRNA gene sequence was analyzed by RDP Classifier (http://rdp.cme.msu.edu/) against the SILVA 119 16S rRNA database using a confidence threshold of 70%. OTUs that reached 97% similarity were used for alpha diversity estimations, which included diversity (Shannon, Simpson), richness (Chao I), and Good’s coverage and rarefaction curve analysis using Mothur (Version 1.30.2; www.mothur.org/).

A heat map was constructed using the gplot package of R software. PCoA was conducted according to the Bray-Curtis distance matrix calculated using OTU information from each sample. To show differences among samples, a cluster tree was generated with the ape package of R using the average method. LEfSe analysis is a metagenomic analysis approach that performs linear discriminant analysis to assess the effective size of each differentially abundant taxon or OTU; the cladogram is displayed according to effective size.

### Statistical Analysis

Metastats^33^ and the Mann-Whitney test were performed using R software and python scripts, respectively. Student’s *t*-test was performed using SPSS version 20 for Windows.

## Additional Information

**How to cite this article**: Lu, Y. *et al.* Mucosal adherent bacterial dysbiosis in patients with colorectal adenomas. *Sci. Rep.* **6**, 26337; doi: 10.1038/srep26337 (2016).

## Supplementary Material

Supplementary Information

## Figures and Tables

**Table 1:** Summary of pyrosequencing data.

Group	Samples	Reads	OTUs	ACE	Chao I	Shannon	Simpson
Colorectal adenoma patients	Adenomatous Tissues (N = 31)	13878 ± 2530	245 ± 59***	344 ± 91***	327 ± 82***	2.98 ± 0.44**	0.17 ± 0.07**
	Adjacent Tissues (N = 31)	13932 ± 2539	256 ± 59***	361 ± 90***	344 ± 82***	3.02 ± 0.44***	0.15 ± 0.07***
Healthy volunteers	Control Tissues (N = 20)	13838 ± 2539	154 ± 61	198 ± 94	192 ± 86	2.50 ± 0.45	0.23 ± 0.08

Note: The OTU, Chao I, Shannon, and Simpson data were calculated at 3% distance.

**Table 2:** Characteristics of study subjects.

Characteristics	Case (n = 31)	Normal (n = 20)	P-value
Age (years)	53.95 ± 6.1	52.95 ± 5.3	>0.05
Male/female	12/19	9/11	>0.05
BMI (kg/m^2^)	23.2 ± 2.8	21.5 ± 4.3	>0.05
