# Supplementary material for: Mucosal adherent bacterial dysbiosis in patients with colorectal adenomas
Source: Sci Rep. 2016 May 19;6:26337. doi: 10.1038/srep26337 (PMC4872055; doi:10.1038/srep26337)
Supplement: Supplementary Information [file srep26337-s1.pdf]

Mucosal adherent bacterial dysbiosis in patients with colorectal adenomas

Yingying Lu[1*], Jing Chen[1*], Junyuan Zheng[1], Guoyong Hu[1], Jingjing Wang[1], Chunlan Huang[1], Lihong Lou[2], Xingpeng Wang[1], Yue Zeng[1]

[1]Department of Gastroenterology, Shanghai general Hospital, Shanghai JiaoTong University School of Medicine, Shanghai, 200080, China

[2]International Medical Care Center, Shanghai general Hospital, Shanghai JiaoTong University School of Medicine, Shanghai, 200080, China.

Corresponding author: Yue Zeng, Department of Gastroenterology, Shanghai general Hospital, Shanghai JiaoTong University School of Medicine, Shanghai, 200080, China. E-mail: zengyue1592@yahoo.com. Tel: 86-21-37798576.

1 **Supplementary Figure Legends**

2 **Figure S1** Rarefaction curve of single sample in each groups (n=3). X-axis means sequencing reads from each sample. Y-axis means the average number of OTUs (a cutoff of 0.03) that were observed for that sampling intensity based on the number of iterations, which is 1,000 by default in muthor.

3 **Figure S2** Shannon curve of single sample in each groups (n=3). X-axis means sequencing reads from each sample. Y-axis means the Shannon diversity index for an OTU definition of 0.03

4 **Figure S3** Venn diagram for each OTU definition of 0.03 to compare the richness shared among three groups.

5 **Figure S4** Non-metric multidimensional scaling plot of mucosal bacterial community structure between healthy controls and colorectal adenomas.

6 **Figure S1**

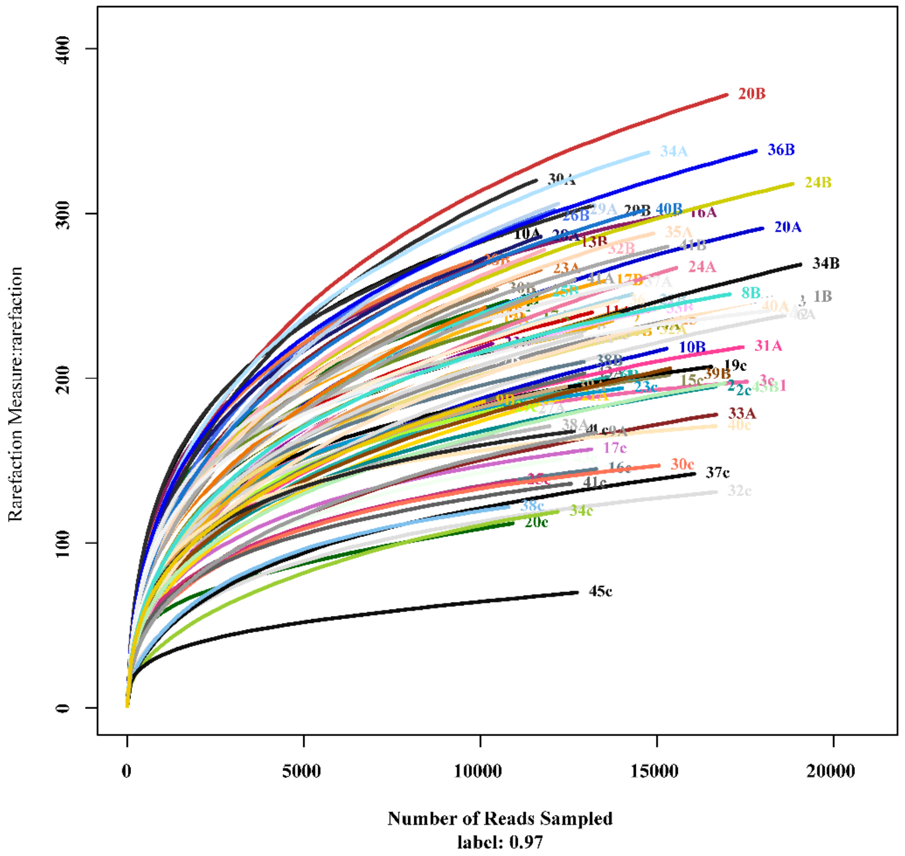
Rarefaction Measure:rarefaction
0
100
200
300
400
0
5000
10000
15000
20000
Number of Reads Sampled
label: 0.97
20B
34A
36B
24B
30A
20A
34B
24A
8B
1B
10B
31A
19c
33A
17c
30c
37c
32c
38c
20c
34c
45c

Figure S2

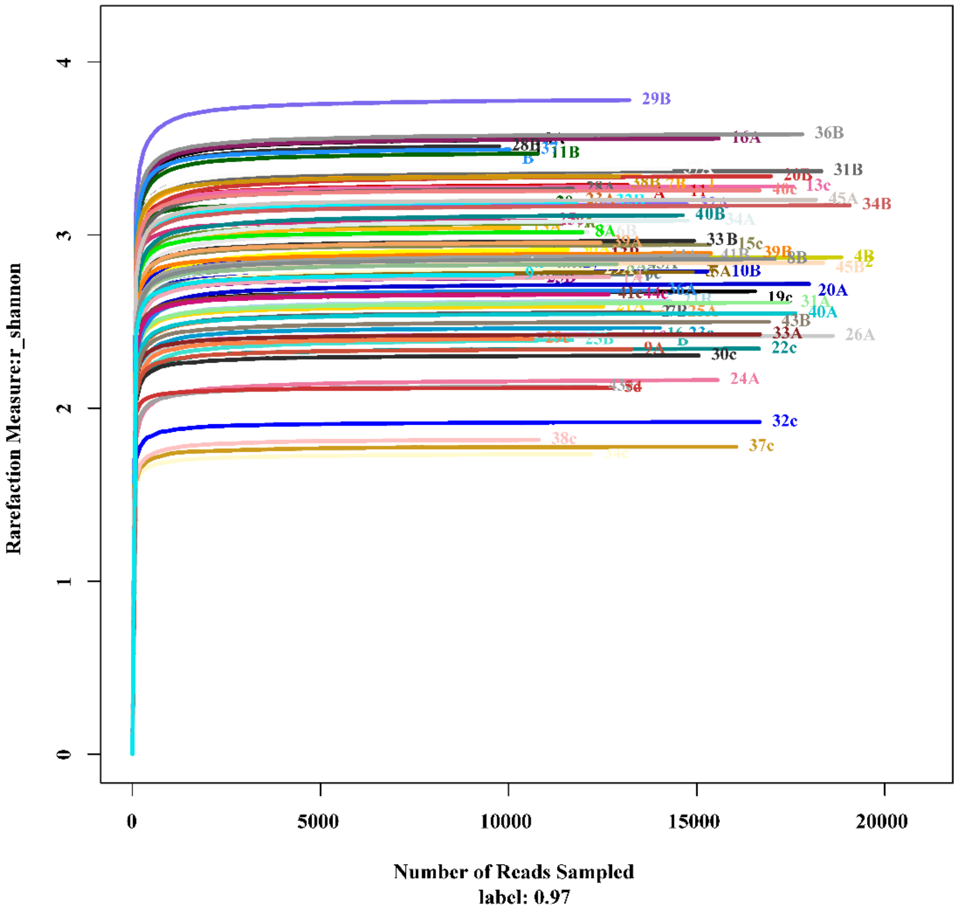
Rarefaction Measure:r_shannon
0
1
2
3
4
0
5000
10000
15000
20000
Number of Reads Sampled
label: 0.97
29B
36B
16A
11B
31B
20B
34B
40B
33B
39B
4B
10B
20A
19c
31A
40A
43B
33A
26A
22c
30c
24A
32c
38c
37c

Figure S3

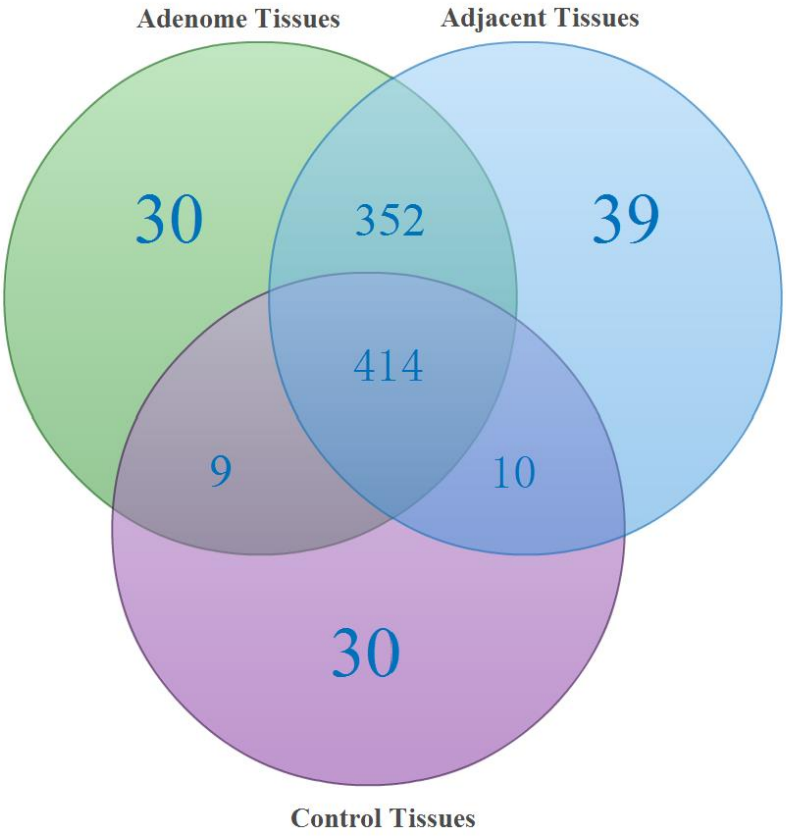

Figure S4

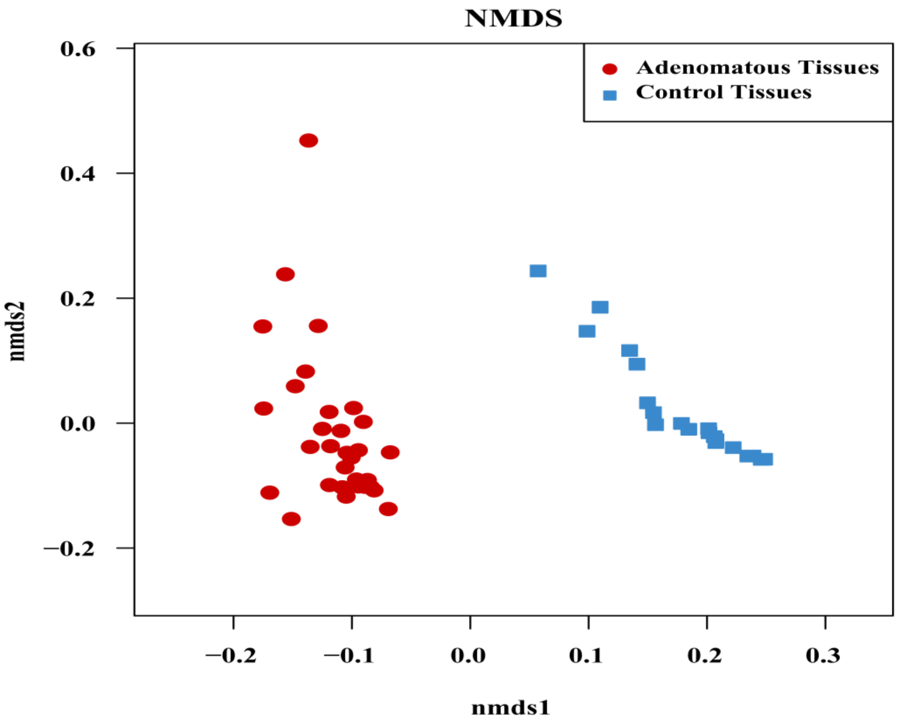

Supplementary Table

Table S1 Pyrosequencing data and estimator index of each sample

Sample ID	Reads	0.97					
		OTU	ace	chao	coverage	shannon	simpson
10A	10605	287	358 332,401	354 324,409	0.993116	3.55 (3.51,3.59)	0.1042 (0.0994,0.109)
10B	15275	218	304 271,358	314 271,394	0.995155	2.79 (2.76,2.82)	0.1613 (0.1563,0.1663)
11A	13164	240	383 343,438	329 288,406	0.994682	3.29 (3.26,3.32)	0.1097 (0.1057,0.1137)
11B	10778	247	325 295,375	331 292,405	0.993691	3.47 (3.44,3.51)	0.0936 (0.0897,0.0975)
13A	10279	235	404 360,463	340 294,424	0.992217	3.04 (3,3.08)	0.15 (0.1439,0.1561)
13B	11726	282	375 342,425	365 330,426	0.992666	2.89 (2.85,2.92)	0.1975 (0.1904,0.2046)
13c	17553	198	220 209,242	220 207,251	0.998234	3.28 (3.26,3.31)	0.0967 (0.0938,0.0996)
15c	15344	202	245 226,279	247 223,296	0.997002	2.94 (2.91,2.97)	0.1601 (0.1548,0.1653)
16A	15579	300	361 338,399	350 327,392	0.995764	3.56 (3.53,3.59)	0.0908 (0.0875,0.0942)
16B	13618	201	332 295,386	279 243,346	0.994933	2.43 (2.39,2.46)	0.2347 (0.2278,0.2416)
16c	13280	145	187 167,225	176 159,213	0.997139	2.77 (2.74,2.8)	0.1322 (0.1285,0.1358)
17A	11420	237	301	296	0.994396	3.07	0.1405

			276,342	269,348		(3.03,3.1)	(0.135,0.146)
17B	13501	259	390 353,442	357 313,435	0.994297	3.34 (3.31,3.37)	0.0786 (0.0765,0.0808)
17c	13136	157	188 173,218	192 172,236	0.997183	2.43 (2.39,2.46)	0.2423 (0.2351,0.2495)
19c	16543	207	244 227,274	243 224,284	0.997401	2.67 (2.64,2.71)	0.2258 (0.2194,0.2322)
20A	17981	291	396 360,450	391 349,462	0.994939	2.72 (2.69,2.75)	0.2168 (0.2107,0.2228)
20B	16968	372	486 450,539	470 433,532	0.993517	3.34 (3.31,3.37)	0.1205 (0.1164,0.1247)
20c	10920	112	270 221,343	194 147,308	0.996429	3.18 (3.16,3.21)	0.064 (0.0623,0.0657)
21A	12507	189	275 240,331	270 233,341	0.994563	2.59 (2.55,2.62)	0.1921 (0.1858,0.1983)
21B	14297	251	329 300,373	342 302,413	0.994474	2.62 (2.59,2.65)	0.2099 (0.2035,0.2162)
22c	16653	195	254 229,296	268 232,338	0.996577	2.34 (2.31,2.37)	0.2704 (0.2635,0.2773)
23A	11710	266	353 322,403	349 313,412	0.992997	3.2 (3.17,3.23)	0.1071 (0.1035,0.1107)
23B	10335	221	362 323,415	287 258,339	0.99284	2.75 (2.71,2.79)	0.1653 (0.16,0.1706)
23c	14014	194	229 213,258	233 212,279	0.997074	2.46 (2.43,2.5)	0.2561 (0.2487,0.2635)
24A	15549	267	369 334,422	353 318,411	0.993826	2.16 (2.13,2.19)	0.3039 (0.2963,0.3115)
24B	18841	318	411 379,46	407 370,46	0.995117	2.87 (2.84,2.9)	0.1891 (0.1838,0.1945

			0	9			)
25A	14101	242	346 309,406	339 297,412	0.994043	2.55 (2.52,2.59)	0.2184 (0.2119,0.2249)
25B	11687	252	335 305,381	316 289,365	0.993155	2.39 (2.35,2.43)	0.2719 (0.2635,0.2803)
25c	10991	138	176 158,211	170 152,210	0.996725	3.08 (3.06,3.11)	0.1026 (0.0986,0.1067)
26A	18614	238	316 287,362	304 275,356	0.995971	2.42 (2.39,2.45)	0.2392 (0.2332,0.2452)
26B	12094	302	397 364,446	393 356,456	0.992228	3.02 (2.98,3.05)	0.1632 (0.1572,0.1692)
27A	10701	181	227 206,263	230 204,285	0.995701	2.88 (2.85,2.92)	0.1444 (0.1397,0.1492)
27B	13734	235	339 301,398	346 298,429	0.993738	2.55 (2.52,2.59)	0.2104 (0.204,0.2167)
28A	11710	286	392 355,450	436 371,548	0.992143	3.27 (3.23,3.3)	0.1305 (0.1253,0.1358)
28B	9743	271	350 321,395	332 305,381	0.992405	3.51 (3.48,3.55)	0.1004 (0.0956,0.1052)
29A	12203	306	407 373,458	391 357,448	0.991887	2.8 (2.76,2.84)	0.1953 (0.1887,0.202)
29B	13204	305	396 363,447	417 369,504	0.993638	3.78 (3.75,3.81)	0.0624 (0.06,0.0648)
29c	10631	182	226 207,260	227 204,275	0.995485	2.4 (2.36,2.44)	0.2639 (0.2553,0.2726)
30A	11574	320	442 403,501	419 380,483	0.990928	2.92 (2.88,2.96)	0.1931 (0.1861,0.2001)
30B	10462	254	351 316,404	337 302,398	0.991875	2.83 (2.8,2.87)	0.185 (0.1781,0.192)

30c	15052	147	172 159,197	165 154,190	0.99794	2.3 (2.27,2.33)	0.266 (0.2588,0.2732)
31A	17432	219	273 251,309	265 243,308	0.996615	2.61 (2.58,2.64)	0.1843 (0.1793,0.1893)
31B	18307	249	352 313,416	363 312,455	0.995685	3.37 (3.34,3.39)	0.0822 (0.0798,0.0847)
32A	14733	229	370 331,424	317 277,391	0.995113	3.18 (3.15,3.2)	0.0934 (0.0906,0.0962)
32B	11801	278	445 402,502	387 342,463	0.99195	3.19 (3.16,3.23)	0.1223 (0.1175,0.1271)
32c	16669	131	165 148,198	162 145,203	0.9979	1.92 (1.89,1.95)	0.338 (0.3303,0.3457)
33A	16678	178	243 216,290	268 223,357	0.996462	2.43 (2.4,2.45)	0.2156 (0.2098,0.2214)
33B	14925	242	310 283,353	296 271,342	0.995444	2.97 (2.94,3)	0.1239 (0.1202,0.1277)
34A	14758	337	449 412,505	447 403,520	0.993156	3.08 (3.05,3.12)	0.1696 (0.1639,0.1752)
34B	19066	269	529 468,610	444 369,576	0.994965	3.17 (3.15,3.2)	0.0955 (0.0935,0.0976)
34c	12191	119	163 142,202	148 132,182	0.996719	1.73 (1.7,1.76)	0.3659 (0.3568,0.375)
35A	14892	288	386 352,437	369 335,428	0.994091	2.79 (2.75,2.82)	0.2049 (0.1986,0.2113)
35B	12939	228	268 250,299	271 249,317	0.99629	3.38 (3.35,3.41)	0.0992 (0.0954,0.1029)
36A	13878	247	408 365,466	338 298,409	0.994308	2.68 (2.64,2.72)	0.2349 (0.2275,0.2422)
36B	17802	338	453	464	0.994326	3.58	0.077

			414,510	413,550		(3.56,3.61)	(0.0745,0.0796)
37A	14307	258	377<br>343,425	329<br>296,391	0.995317	3.38<br>(3.35,3.41)	0.0917<br>(0.0886,0.0948)
37B	10025	211	269<br>244,312	297<br>253,384	0.994414	3.49<br>(3.46,3.53)	0.0649<br>(0.0628,0.067)
37c	16050	142	189<br>168,227	187<br>163,237	0.997196	1.78<br>(1.75,1.8)	0.3685<br>(0.3604,0.3766)
38A	11959	171	221<br>199,262	216<br>192,265	0.996154	3.02<br>(2.99,3.05)	0.1078<br>(0.1046,0.111)
38B	12930	210	308<br>277,353	289<br>250,365	0.995514	3.34<br>(3.31,3.37)	0.0761<br>(0.0738,0.0784)
38c	10799	122	149<br>135,175	150<br>134,187	0.996944	1.82<br>(1.78,1.85)	0.3558<br>(0.3462,0.3653)
39A	12433	195	258<br>232,303	272<br>235,345	0.995174	2.96<br>(2.92,2.99)	0.1437<br>(0.1384,0.149)
39B	15376	206	294<br>259,350	299<br>256,379	0.995447	2.89<br>(2.87,2.92)	0.1339<br>(0.1296,0.1382)
40A	17624	246	332<br>301,381	321<br>289,379	0.995461	2.55<br>(2.52,2.58)	0.2031<br>(0.1977,0.2085)
40B	14623	302	374<br>348,416	369<br>341,418	0.994187	3.11<br>(3.08,3.15)	0.1386<br>(0.1338,0.1434)
40c	16666	171	193<br>181,216	195<br>180,231	0.99832	3.26<br>(3.23,3.28)	0.0985<br>(0.0952,0.1018)
41A	12673	260	494<br>438,567	398<br>341,493	0.992109	2.76<br>(2.72,2.79)	0.1753<br>(0.1699,0.1808)
41B	15295	280	354<br>327,398	355<br>323,413	0.99477	2.88<br>(2.85,2.91)	0.1758<br>(0.1703,0.1813)
41c	12563	136	172<br>155,20	166<br>149,20	0.997214	2.66<br>(2.63,2.69	0.185<br>(0.1787,0.1914

			7	5		)	)
43A	12952	203	354<br>312,412	292<br>251,369	0.994595	2.85<br>(2.82,2.88)	0.1608<br>(0.1552,0.1664)
43B	16918	197	308<br>274,357	258<br>229,313	0.996335	2.5<br>(2.47,2.53)	0.2293<br>(0.2231,0.2355)
43c	12310	151	187<br>170,219	182<br>165,219	0.996751	2.12<br>(2.09,2.16)	0.3031<br>(0.2947,0.3116)
44A	13913	239	323<br>291,373	329<br>288,403	0.994681	2.87<br>(2.84,2.91)	0.1838<br>(0.1776,0.1901)
44B	10127	243	479<br>423,552	359<br>310,441	0.990817	2.83<br>(2.79,2.87)	0.1783<br>(0.1715,0.1852)
44c	12655	168	202<br>186,234	201<br>183,243	0.997076	2.66<br>(2.62,2.69)	0.2133<br>(0.2062,0.2203)
45A	18166	241	274<br>259,302	277<br>258,318	0.997578	3.2<br>(3.17,3.23)	0.1375<br>(0.133,0.142)
45B	18353	249	313<br>288,354	321<br>287,383	0.996404	2.84<br>(2.81,2.87)	0.1759<br>(0.1709,0.1809)
45c	12733	70	136<br>109,181	116<br>86,202	0.998115	2.12<br>(2.09,2.14)	0.2099<br>(0.2046,0.2152)
8A	12872	226	361<br>323,414	333<br>284,422	0.994173	2.83<br>(2.8,2.87)	0.1788<br>(0.1726,0.1851)
8B	17064	251	306<br>284,341	305<br>280,353	0.996249	2.86<br>(2.83,2.89)	0.1737<br>(0.1684,0.179)
9A	13260	167	229<br>203,275	216<br>192,265	0.996003	2.34<br>(2.31,2.37)	0.24<br>(0.2329,0.2471)
9B	10104	186	344<br>298,408	290<br>241,382	0.99327	2.77<br>(2.73,2.81)	0.1693<br>(0.163,0.1757)
